# Automatic Calibration of an Around View Monitor System Exploiting Lane Markings

**DOI:** 10.3390/s18092956

**Published:** 2018-09-05

**Authors:** Kyoungtaek Choi, Ho Gi Jung, Jae Kyu Suhr

**Affiliations:** 1 Department of Electronic Engineering, Korea National University of Transportation, 50 Daehak-ro, Chungju-si, Chungbuk 27469, Korea; maninquestion75@gmail.com (K.C.); hogijung@ut.ac.kr (H.G.J.); 2 School of Intelligent Mechatronics Engineering, Sejong University, 209 Neungdong-ro, Gwangjin-gu, Seoul 05006, Korea

**Keywords:** around view monitor (AVM) system, automatic calibration, lane marking, parking assist system, advanced driver assistance system (ADAS)

## Abstract

This paper proposes a method that automatically calibrates four cameras of an around view monitor (AVM) system in a natural driving situation. The proposed method estimates orientation angles of four cameras composing the AVM system, and assumes that their locations and intrinsic parameters are known in advance. This method utilizes lane markings because they exist in almost all on-road situations and appear across images of adjacent cameras. It starts by detecting lane markings from images captured by four cameras of the AVM system in a cost-effective manner. False lane markings are rejected by analyzing the statistical properties of the detected lane markings. Once the correct lane markings are sufficiently gathered, this method first calibrates the front and rear cameras, and then calibrates the left and right cameras with the help of the calibration results of the front and rear cameras. This two-step approach is essential because side cameras cannot be fully calibrated by themselves, due to insufficient lane marking information. After this initial calibration, this method collects corresponding lane markings appearing across images of adjacent cameras and simultaneously refines the initial calibration results of four cameras to obtain seamless AVM images. In the case of a long image sequence, this method conducts the camera calibration multiple times, and then selects the medoid as the final result to reduce computational resources and dependency on a specific place. In the experiment, the proposed method was quantitatively and qualitatively evaluated in various real driving situations and showed promising results.

## 1. Introduction

**Figure 1:**
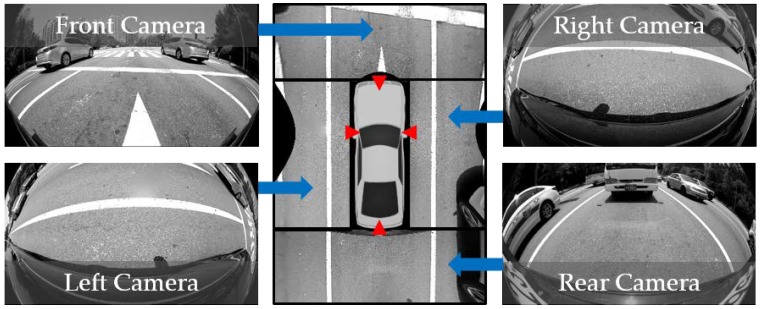
Camera configuration of the around view monitor (AVM) system. Red triangles indicate four cameras of the AVM system.

**Figure 2:**
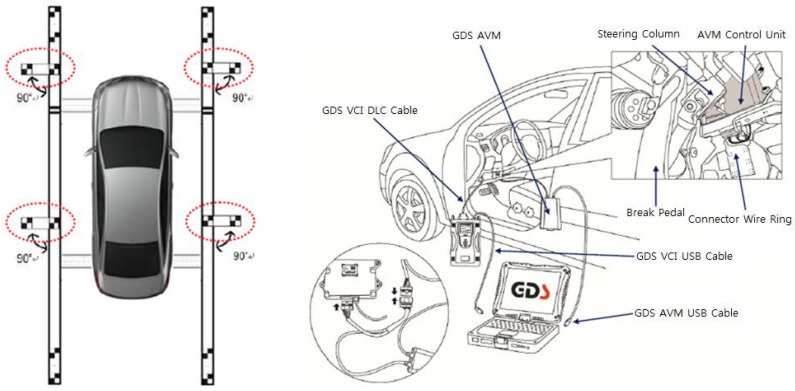
Example of the calibration pattern (**left**) and external equipment (**right**) used for recalibrating the AVM system [3].

Recently, around view monitor (AVM) systems have become popular as parking aid products because they provide convenience to drivers by showing the surrounding view of the vehicle [1]. The AVM system consists of four cameras located at the centers of the front and rear bumpers and under two side view mirrors as shown in Figure 1 with red triangles [2]. Four images acquired from four cameras are transformed into bird’s eye view images, and they are stitched to generate an AVM image. To this end, intrinsic and extrinsic parameters of four cameras should be known. The intrinsic parameters describe the optical properties of the camera, and the extrinsic parameters describe the relationship between the camera and vehicle coordinate systems. Since the intrinsic parameters hardly change, they need to be calibrated only once when the camera is manufactured. On the other hand, the extrinsic parameters can be changed due to various external shocks so that they need to be occasionally calibrated. However, the recalibration is quite inconvenient because, in reality, it should be carried out by skilled workers with a calibration pattern and external equipment as shown in Figure 2. This means that drivers must visit large-scale repair shops equipped with those specialized facilities.

To alleviate this inconvenience, an automatic calibration method for AVM systems should be developed. In order for an automatic calibration method to be practical and useful, the following points should be considered. First, it should use visual features that are easily observable in typical road conditions. Second, it should operate at various vehicle speeds that can occur in natural driving situations. Last, it should consider the relationship between adjacent cameras in order to obtain a seamless AVM image. Many methods have been suggested for calibrating vehicle-mounted cameras, and they can be categorized into three approaches: calibration pattern-based, interest point-based, and lane marking-based. The calibration pattern-based approach is inconvenient, because it needs specialized patterns. The interest point-based approach is limited by the vehicle speed. Since four cameras of the AVM system are facing the ground at low heights as shown in Figure 1, point features move too fast to reliably track when the vehicle travels at high speed. In addition, it is difficult to obtain point correspondences between images of adjacent cameras because overlapping areas are severely distorted due to the use of fisheye lenses as shown in Figure 1. On the other hand, the lane marking-based approach is suitable for calibrating the AVM system since lane markings exist in almost all on-road situations and are detectable at both low and high speeds. Furthermore, it is easier to find corresponding lane markings in images of adjacent cameras compared with the point features. However, previous methods in the lane marking-based approach only deal with a single camera and assume that the lane markings are correctly detected.

**Figure 3:**
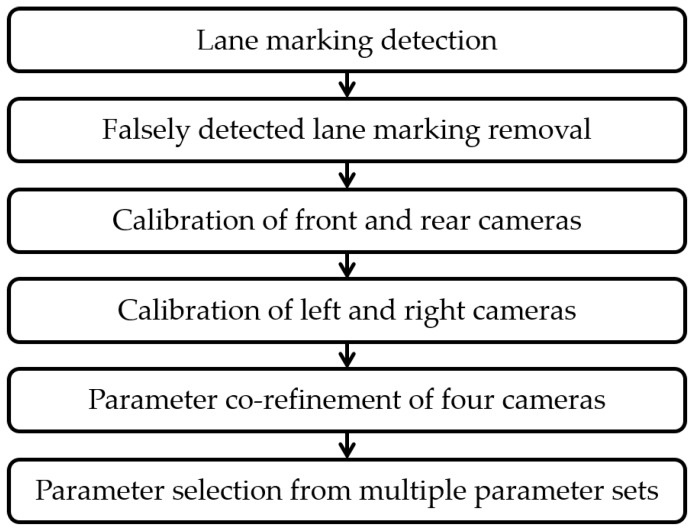
Flowchart of the proposed method.

Based on these analyses, this paper proposes a novel and practical method that can automatically calibrate four cameras of the AVM system using lane markings. The proposed method assumes that four cameras are mounted on the vehicle as shown in Figure 1. Unlike the previous methods, the proposed method calibrates multiple cameras by automatically finding corresponding lane markings across images of adjacent cameras and efficiently handling falsely detected lane markings. Since the camera orientation more severely degrades the quality of the AVM image compared with the camera location [4], this paper focuses on estimating the rotation angles of four cameras. The proposed method starts by detecting lane markings from images captured by four cameras of the AVM system in a cost-effective manner. Since the detection results inevitably include falsely detected lane markings, it identifies and rejects those outliers by utilizing the statistical properties of the detected lane markings. Once the correct lane markings are sufficiently gathered, this method first calibrates the front and rear cameras, and then calibrates the left and right cameras with the help of the calibration results of the front and rear cameras. This two-step approach is essential because side cameras cannot be fully calibrated by themselves, due to insufficient lane marking information. After this initial calibration, this method collects the corresponding lane markings appearing across images of adjacent cameras and simultaneously refines the initial calibration results of four cameras to obtain seamless AVM images. In the case of a long image sequence, this method conducts the above procedure multiple times by dividing the image sequence, and selects the medoid of the multiple calibration results as the final one to reduce both computational resources and dependency on a specific place. Figure 3 shows the flowchart of the proposed method.

The rest of this paper is organized as follows. Section 2 briefly explains related research. Section 3, Section 4, Section 5 and Section 6 describe the essential procedures of the proposed method: lane marking detection, false detection removal, parameter estimation, and parameter selection. Section 7 presents experimental results and analyses. Finally, this paper is concluded with a summary in Section 8.

## 2. Related Research

Since camera calibration is a subject that has been extensively researched for a long period of time, it is unreasonable to cover all the aspects in this paper. Thus, this literature review focuses only on the previous methods suggested for calibrating extrinsic parameters of vehicle-mounted cameras. According to which features are used, the previous methods can be categorized into three approaches: calibration pattern-based, interest point-based, and lane marking-based.

### 2.1. Calibration Pattern-Based Approach

The calibration pattern-based approach estimates camera parameters using special patterns that consist of corners, circles, or lines. Since this approach uses precisely drawn patterns whose configurations are known, it is possible to accurately estimate the camera parameters. However, the methods in this approach are quite inconvenient, because drivers must prepare those patterns themselves, or visit repair shops that can equip those patterns. Chang et al. [5] utilized a pattern composed of multiple rectangles drawn on the ground. This method compares images of rectangles with the reference rectangles to estimate extrinsic parameters of four cameras composing the AVM system. Mazzei et al. [6] used a checkerboard pattern on the ground to calibrate extrinsic parameters of the front view camera by minimizing the reprojection error of the corner locations. Hold et al. [7] used a similar approach using a pattern of circles on the ground. This method finds extrinsic parameters of the front view camera that minimizes the reprojection error of the centers of the circles. Lebraly et al. [8] utilized a pattern composed of multiple circles and dots to estimate the relative pose between rigidly coupled cameras based on the hand–eye calibration scheme and bundle adjustment. Antonelli et al. [9] calibrated a mobile robot mounted camera by utilizing a rectangular parallelepiped pattern, with multiple dots and odometry calculated by wheel encoders. Tan et al. [10] utilized an H-shaped pattern that consisted of three lines (two are parallel and one is perpendicular to the vehicle) to calibrate the front view camera. Li et al. [11] also used an H-shaped pattern to calibrate a rear view camera with a fisheye lens. This method exploits the pattern embedded in parking lot lines. Natroshvili et al. [12] estimated the rotations of four cameras composing the AVM system using point patterns. This paper presents several calibration approaches that use different configurations of the point patterns.

### 2.2. Interest Point-Based Approach

The interest point-based approach estimates the camera parameters using the interest points extracted and tracked from consecutive images. If the interest points are reliably extracted and tracked, this approach produces accurate calibration results. However, in terms of the AVM system calibration, this approach has several drawbacks. Interest points are hardly tracked through the image sequence when the vehicle moves fast, because the cameras of the AVM system are facing the ground at low heights as shown in Figure 1. It is also difficult to obtain point correspondences between images of adjacent cameras because overlapping areas are severely distorted due to the use of fisheye lenses as shown in Figure 1. This makes it difficult to consider the relationships between adjacent cameras to obtain seamless AVM images. Since this approach is not well suited for AVM systems, this literature review introduces some which are closely related with this paper. Miksch et al. [13] calibrated the orientation of the camera attached to the sideview mirror by using homography and vehicle odometry. They reduce the number of parameters of the homography into one with the help of the vehicle odometry, and estimate them by minimizing the brightness difference around the point correspondences between two consecutive images. They proposed a similar method [14] that uses two point correspondences to estimate the homography. This method assumes that the vehicle moves straight ahead to remove the necessity of the vehicle odometry. Ruland et al. [4] estimated the orientation of the fisheye camera by using homography and vehicle odometry. This method finds the camera’s rotation angles that minimize the pixel displacement error between the points transformed by the homography and the points transformed by the vehicle odometry. Tan et al. [15] calibrated a front view camera by combining optical flow, motion stereo, visual odometry, and vehicle odometry. This method estimates the camera’s extrinsic parameters by comparing the visual odometry and vehicle odometry. The optical flow-based vanishing point and motion stereo-based 3D points are used as constraints. Chao et al. [16] estimated the relation between odometer and camera using a two-step least square minimization. This method first estimates two orientation parameters and then calculates the remaining parameters based on integrated odometry measurements and point correspondences between consecutive images. Heng et al. [17] calibrated extrinsic parameters between a rig with multiple cameras and vehicle odometry. This method first estimates the camera-odometry transformation for each camera using the tracked interest points, and then optimizes the initial parameters using bundle adjustment. Heng et al. [18] estimated extrinsic parameters of a multicamera system by using a pre-acquired highly accurate map. It finds the camera poses based on the 2D–3D correspondences between each set of synchronized camera images and the map.

### 2.3. Lane Marking-Based Approach

The lane marking-based approach estimates the camera parameters using lane markings on the ground. Lane markings exist in almost all on-road situations and are detectable at both low and high speeds. However, this approach cannot work on off-road situations where lane markings are not presented, and its accuracy can be degraded when lane markings are worn. Hold et al. [19] calibrated extrinsic parameters of the front view camera. This method detects dashed lane markings at predefined vertical coordinates, and calculates the extrinsic parameters by analyzing the detected lane markings and the measured vehicle velocity. Paula et al. [20] estimated the height, pitch, and roll of the front view camera using a rectangular portion of the road, which is calculated from the lane boundaries and the distance between adjacent dashed lane markings. Wang et al. [21] estimated the extrinsic parameters of the front view camera based on two vanishing points generated from dashed lane markings. One vanishing point is obtained from left and right lane markings and the other one is obtained from lines connecting the corners of the lane segments. These three methods [19,20,21] require dashed lane markings, and two of them [20,21] need accurate vehicle speed synchronized with the camera. Ribeiro et al. [22] proposed a method similar to the method in [20]. This method finds a trapezoid composed with two lane markings and estimates the parameters which transform the trapezoid to a rectangle. Xu et al. [23] calibrated the extrinsic parameters of the front view camera using parallel lines extracted from lane markings, as well as parallel lines perpendicular to the ground (e.g., edges of buildings). This method manually designates lane markings and requires parallel lines perpendicular to the ground, which are rarely captured by the ground-facing cameras of the AVM system. Catala-Prat et al. [24] calibrated the orientation of the front view camera by finding parameters that minimize the sum of squares of the lane markings’ slopes. This method automatically detects lane markings, but manually rejects outliers for reliable parameter estimation. Zhao et al. [25] estimated the pitch and yaw of the vehicle mounted camera. This method uses a vanishing point calculated by applying the weighted least squares approach to detected lane markings. The calculated vanishing point is tracked by Kalman filter to obtain consistency.

As aforementioned in the introduction, the lane marking-based approach is more suitable for calibrating the AVM system in natural driving conditions compared to the other approaches. This is because of the following reasons: lane markings exist in almost all on-road situations, are detectable at both low and high speeds, and appear across images of adjacent cameras. However, previous methods in this approach cannot be directly used to calibrate cameras of the AVM system because they require ideally drawn dashed lanes, both left and right lanes at the same time, or lines perpendicular to the ground. Furthermore, previous methods have not handled falsely detected lane markings that are inevitably included in a real situation, and have not utilized the lane markings appearing across images of adjacent cameras.

In order to overcome the limitations of the previous methods, this paper proposes a novel lane marking-based automatic calibration method for AVM systems. The proposed method can handle both dashed and solid lane markings, calibrate side view cameras where only the left or right lane marking is observed, and does not need rare objects, such as lines perpendicular to the ground. In addition, this method explicitly deals with falsely detected lane markings based on the statistical properties of the detected lane markings, and utilizes the lane markings that appear across images of adjacent cameras to consider the inter-camera relationships.

Besides these three approaches, there is a road geometry-based approach that calibrates vehicle-mounted cameras by estimating the geometry of the road in front of the vehicle [26,27]. Since almost all methods in this approach use stereo cameras to obtain three-dimensional geometry information of the road, it cannot be utilized for calibrating the cameras of the AVM system where there is very little overlap between views of adjacent cameras. Therefore, this literature review does not deal with this approach in depth.

## 3. Lane Marking Detection

**Figure 4:**
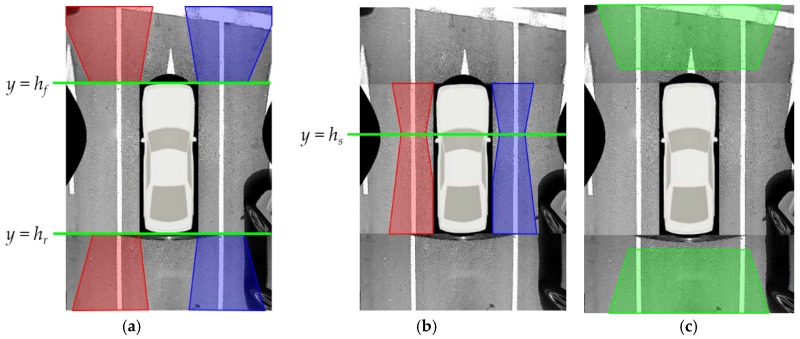
Precalculated position range of lane markings. (**a**) Position ranges of left and right lanes in images of the front and rear cameras; (**b**) Position ranges of left and right lanes in images of the left and right cameras; (**c**) Position ranges of stop lines in images of the front and rear cameras. Red, blue, and green regions indicate the position ranges of left lanes, right lanes, and stop lines, respectively.

The proposed method detects lane markings by combining simple techniques: edge detection, line estimation, and line pairing. This paper deliberately avoids using sophisticated lane detection methods in order to detect lane markings from images of four cameras in real-time. This method assumes that the angles of the four cameras can be changed within ±5°. This value was empirically set with the help of automotive experts. This method precalculates the ranges of position, orientation, and width for lane markings as geometrical constraints during offline simulation. Figure 4 shows the precalculated position range of the lane markings in the AVM image. Figure 4a–c are the position ranges of the left and right lanes in the images of the front and rear cameras, the left and right lanes in images of the left and right cameras, and the stop lines in the images of the front and rear cameras, respectively. Red, blue, and green regions indicate the position ranges for left lanes, right lanes, and stop lines, respectively. The ranges of orientation and width are not depicted because it is difficult to graphically draw them.

**Figure 5:**
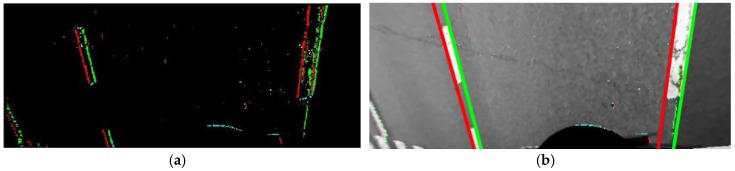
(**a**) Edge detection and classification result (red: rising edge pixels, green: falling edge pixels); (**b**) Lane marking detection result (red: lines from rising edge pixels, green: lines from falling edge pixels).

**Figure 6:**
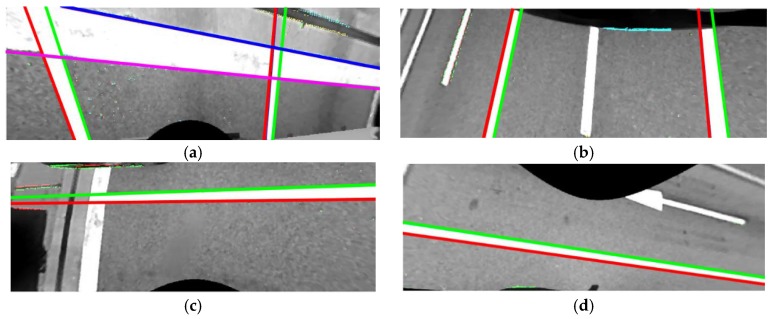
Example detection results of lane marking and stop line. (**a**) Two lanes and a stop line detected in a front camera image; (**b**) Two lanes detected in a rear camera image; (**c**) A left lane detected in a left camera image; (**d**) A right lane detected in a right camera image.

The proposed method detects lane markings in bird’s-eye view images. Since the angles of the cameras have not yet been calibrated, the bird’s-eye view images can erroneously be generated with the initial camera angles. However, it is easier to detect lane markings in bird’s-eye view images compared to undistorted images because they are less affected by perspective distortion. Before detecting lane markings, a 3 × 3 median filter is applied to the bird’s-eye view image to remove salt and pepper-like noises that frequently appear on asphalt roads. The image gradient is calculated by the Sobel operator [28] and pixels whose gradient magnitudes are larger than a predetermined value are detected as edge pixels. Each edge pixel is classified according to its gradient orientation. If an edge pixel has a gradient orientation between −90° and 90°, it is classified as a rising edge pixel. Otherwise, it is classified as a falling edge pixel. Figure 5a shows an edge detection and classification result in a bird’s-eye view image acquired by the front camera of the AVM system. Red and green dots indicate the rising and falling edge pixels, respectively. Once edge pixels are obtained, straight lines are estimated by random sample consensus (RANSAC) [29]. In this procedure, RANSAC is separately applied to the rising and falling edge pixels. If the detected line satisfies the ranges of position and orientation precalibrated during the offline simulation, it is regarded as valid. Otherwise, it is discarded. Figure 5b shows four straight lines estimated by RANSAC using the edge pixels presented in Figure 5a. Red and green lines are estimated from the rising and falling edge pixels, respectively. Once the lines are estimated, they are paired to compose lane markings based on three conditions. The lines that follow the conditions are used for further calibration procedures, and the lines that do not follow the conditions are discarded. The three conditions are as follows: (1) Two lines should not be crossed within the bird’s-eye view image because they are parallel in the real world; (2) The distance between two lines should be within the width range of the lane marking, which is precalculated during the offline simulation; (3) The edge pixels of two lines composing a lane marking should have similar vertical positions. This means that the number of edge pixels whose vertical positions are similar should be larger than a predetermined value. The detection procedure of the stop line is almost the same as that of the lane marking. The only differences are that it uses the precalculated range of the stop line width, and counts the number of edge pixels whose horizontal positions are similar. Figure 6a–d show some examples of the lane marking and stop line detection results. Figure 6a includes two lanes and a stop line detected in a front camera image, Figure 6b includes two lanes detected in a rear camera image, and Figure 6c,d include left and right lanes detected in images of the left and right cameras, respectively.

## 4. Falsely Detected Lane Marking Removal

In real situations, the lane marking detection result inevitably includes false detections, so-called outliers. These outliers should be rejected because they can severely degrade the calibration result. However, the previous methods in the lane making-based approach introduced in Section 2 ignore this important procedure, and simply assume that all lane markings are correctly detected, or manually reject the outliers. Since a fully automated calibration method should include a procedure that handles outliers, this paper suggests a method that identifies and rejects outliers based on the statistical properties of the detected lane markings. The suggested method assumes that the vehicle travels along the lane and that straight driving is more frequent than curved driving. Under these assumptions, this method statistically draws two common properties of the correctly detected lane markings in terms of position and orientation, and then rejects the lane markings that do not follow those properties.

**Figure 7:**
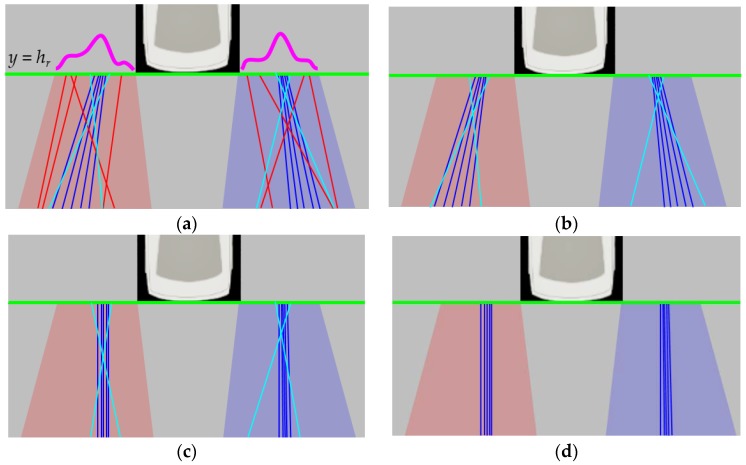
Outlier rejection based on the lane marking position and orientation. (**a**) Two positional histograms (magenta lines); (**b**) Position-based outlier rejection result; (**c**) Lane markings transformed by the vanishing point-based homography; (**d**) Orientation-based outlier rejection result.

This method first finds the common property of lane marking position, and rejects outliers using it. To draw the positional property, this method calculates a positional histogram based on the intersections between the detected lane markings and the line *y* = *h*. A total of six positional histograms are generated for left and right lane markings in the front camera images, left and right lane markings in the rear camera images, left lane markings in the left camera images, and right lane markings in the right camera images. The value of *h* is set to *h_f_* for the lane markings in the front camera images, *h_r_* for the lane markings in the rear camera images, and *h_s_* for the lane markings in left and right camera images. *h_f_*, *h_r_*, and *h_s_* are shown in Figure 4a,b. *h_f_*, *h_r_*, and *h_s_* are selected at the locations where positional variations of the detected lane markings are expected to be small according to the offline simulation. Once six positional histograms are generated, their modes are estimated. If a histogram is multimodal, the mode closest to the vehicle is selected. After obtaining a mode for each histogram, this method rejects the lane markings whose locations at *y* = *h* are farther from the mode by the predetermined value. Figure 7a,b show the position-based outlier rejection procedure in the case of the rear camera image. Blue, red, and cyan lines in Figure 7a show the detected lane markings, and two thick magenta curves indicate two positional histograms for left and right lane markings. Using the highest modes of those two histograms (magenta lines), the red lines can be removed because their locations at *y* = *h_r_* are distant from the corresponding modes. Figure 7b shows the remaining lines after the position-based outlier rejection.

The remaining lane markings after the position-based outlier rejection are filtered, once again, based on their orientations. To this end, this method utilizes the vanishing point and homography. In cases of the lane markings detected in images of the left and right cameras, the vanishing point is estimated by RANSAC. This first randomly selects two lines and calculates their intersection point, **v***_l_* = [*u_p_* *v_p_* 1]^T^. A rotation matrix, *R*, that transforms **v***_l_* into a point at infinity, **p***_∞_* = [0 1 0]^T^, is obtained by calculating the rotation axis, **u** and angle, *θ* as

(1) $$\begin{array}{l} u={\bar{v}}_{l}\times p_{\infty },\;\theta =\cos^{-1}\left({\bar{v}}_{l}\cdot p_{\infty }\right)\\ \mathrm{where}\;{\bar{v}}_{l}=v_{l}/\left\|v_{l}\right\| \end{array},$$

where × and **·** indicate the cross and dot products, respectively. The rotation matrix, *R* can be derived by **u** = [*u_x_ u_y_ u_z_*]^T^ and *θ* as

(2) $$R=\left[\begin{array}{ccc} \cos\theta +u_{x}^{2}\left(1-\cos\theta \right) & u_{x}u_{y}\left(1-\cos\theta \right)-u_{z}\sin\theta & u_{x}u_{z}\left(1-\cos\theta \right)+u_{y}\sin\theta \\ u_{y}u_{x}\left(1-\cos\theta \right)+u_{z}\sin\theta & \cos\theta +u_{y}^{2}\left(1-\cos\theta \right) & u_{y}u_{z}\left(1-\cos\theta \right)-u_{x}\sin\theta \\ u_{z}u_{x}\left(1-\cos\theta \right)-u_{y}\sin\theta & u_{z}u_{y}\left(1-\cos\theta \right)+u_{x}\sin\theta & \cos\theta +u_{z}^{2}\left(1-\cos\theta \right) \end{array}\right].$$

Once *R* is obtained, the lane markings, **l** are transformed by the homography, *H* as

(3) $$l^{\prime }=H^{-T}l={\left(KRK^{-1}\right)}^{-T}l,$$

where *K* is a 3 × 3 intrinsic parameters matrix of the virtual camera of the AVM system, that is predetermined when the AVM system is manufactured. After transforming the lane markings via Equation (3), this method counts the number of transformed lane markings, **l′** that are parallel to the heading direction of the vehicle (vertical axis of the AVM image) as a consensus set. This procedure is iterated, and *H_max_* that maximizes the number of consensus set is selected. Finally, the lane markings excluded from the consensus set of *H_max_* are identified as outliers and rejected.

In cases of the lane markings detected in images of the front and rear cameras, the vanishing point is estimated by a voting-based method. If RANSAC is used in these cases, it requires a large amount of computation cost for transforming lane markings and counting consensus sets. This is because the number of lane markings in images of the front and rear cameras are approximately twice that of the side camera. Unlike the side camera, both left and right lane marking can be captured by the front and rear cameras. The locations of the vanishing points calculated from a pair of left and right lane markings are accumulated in 2D voting bins. The final vanishing point is estimated by averaging the locations that have the largest accumulation results. The homography, *H_max_*, is calculated from the final vanishing point using Equations (1)–(3). All lane markings are transformed by *H_max_* using Equation (3), and the transformed lane markings not parallel to the heading direction of the vehicle (vertical axis of the AVM image) are identified as outliers and rejected. Figure 7c,d show the orientation-based outlier rejection procedure in the case of the rear camera image. Blue and cyan lines in Figure 7c show the lane markings transformed by the vanishing point-based homography in Equation (3). In Figure 7c, the cyan lines are identified as outliers, and rejected, because they are not parallel to the heading direction of the vehicle. Figure 7d shows the remaining lines after the orientation-based outlier rejection. The remaining lines after both the position-based and orientation-based outlier rejection procedures are used for further calibration procedures, and the rejected lines from either of these two procedures are discarded. The outlier rejection procedure for stop lines are also conducted by RANSAC. This procedure will be described in detail in Section 5 since it is conducted during the parameter estimation.

## 5. Parameter Estimation

The proposed method first calibrates the front and rear cameras, and then calibrates the left and right cameras with the help of the calibration results of the front and rear cameras. This two-step approach is essential for calibrating cameras of the AVM system because side cameras cannot be fully calibrated by themselves due to insufficient lane marking information. As shown in Figure 1, both left and right lane markings are observable in images of the front and rear cameras, so that rotation angles of those cameras can be fully estimated. However, only either the left or right lane marking is observable in images of the left and right cameras, so that rotation angles of those cameras can only be estimated in part. This is the reason why this paper utilizes the two-step approach. Once four cameras are initially calibrated by the two-step approach, this method simultaneously refines the initial calibration results of four cameras using the corresponding lane markings appearing across images of adjacent cameras.

### 5.1. Calibration of Front and Rear Cameras

**Figure 8:**
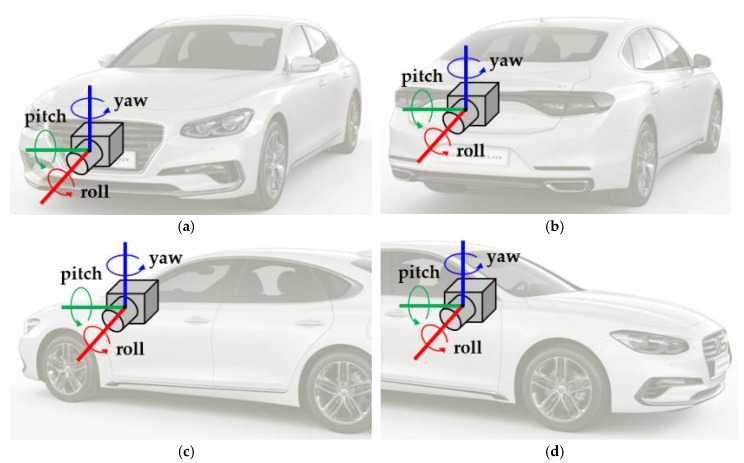
Pitch, yaw, and roll of four cameras composing the AVM system. (**a**) Front camera; (**b**) Rear camera; (**c**) Left camera; (**d**) Right camera.

**Figure 9:**
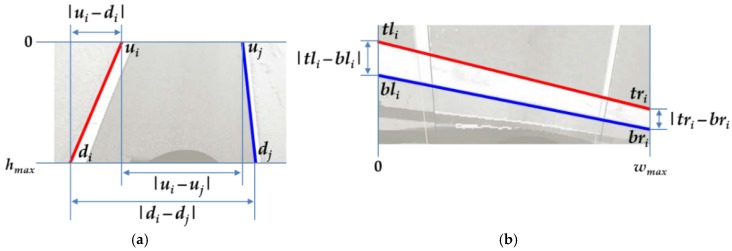
Explanations of cost functions used for the LM algorithm. (**a**) In the case of a lane marking; (**b**) In the case of a stop line.

The proposed method first calibrates the front and rear cameras. Those two cameras are separately calibrated using the same approach. Figure 8a,b show pitch, yaw, and roll of the front and rear cameras, respectively. This method first estimates the pitch and yaw angles of the front and rear cameras, and then estimates their roll angles. The pitch and yaw angles are estimated by finding the vanishing point. If **l***_i_* is a 3 × 1 parameter vector of the *i*-th line survived from the outlier rejection procedure, and **v***_l_* is the vanishing point in homogeneous coordinates, then they should be related as **l***_i_*^T^**v***_l_* = 0. If there are *N_L_* lane markings, it can be expanded as

(4) $$Lv_{l}=0,\;\mathrm{where}\;L={\left[\begin{array}{ccccc} l_{1} & l_{2} & \cdots & l_{2N_{L}-1} & l_{2N_{L}} \end{array}\right]}^{T},$$

where *L* is a (2*N_L_*) × 3 matrix that includes 2*N_L_* lines. There are 2*N_L_* lines in *N_L_* lane markings because each lane marking consists of two lines as shown in Figure 6. Thus, a least-squares solution of **v***_l_* is calculated by finding the eigenvector of *L*^T^*L* corresponding to the smallest eigenvalue. The rotation matrix is obtained from **v***_l_* via Equations (1) and (2), and the pitch and yaw angles are estimated by decomposing the obtained rotation matrix. The roll angle obtained from this rotation matrix may not be correct because the vanishing point does not have enough information to estimate the roll angle. The pitch and yaw angles estimated by this closed-form solution are refined using Levenberg–Marquardt (LM) algorithm [30] by minimizing the cost, *c_py_*, as

(5) $$c_{py}=\sum_{i=1}^{2N_{L}}\left|u_{i}-d_{i}\right|+\sum_{i=1}^{2N_{L}}\sum_{j=1}^{2N_{L}}\left|\left|u_{i}-u_{j}\right|-\left|d_{i}-d_{j}\right|\right|,$$

where *u_i_* and *d_i_* are shown in Figure 9a. They indicate the horizontal locations of the intersection points between the *i*-th line and *y* = 0 and *y* = *h_max_*, respectively. In Equation (5), the left term is minimized when lines are located upright (parallel to the heading direction of the vehicle), and the right term is minimized when pairs of lines are parallel to each other. Thus, *c_py_* is minimized when the lines are both upright and parallel to each other. The vanishing point **v***_l_* is also refined based on the pitch and yaw angles optimized by LM, and the refined vanishing point is denoted by **v***_l_*′.

The one remaining angle, roll, is estimated based on either stop line or lane marking width. If enough stop lines are detected, the roll angle is estimated based on the stop lines. The roll angle estimation and false stop line rejection are simultaneously conducted using RANSAC. A stop line consists of two lines, as shown in Figure 9b with red and blue. A pair of two lines composing a stop line is randomly selected, and the vanishing point, **v***_s_*, is calculated by finding their intersection point. Since the vanishing point induced by the lane markings, **v***_l_*′, is already calculated, the complete rotation matrix that contains all pitch, yaw, and roll angles can be obtained as

(6) $$R=\left[\begin{array}{ccc} {v^{\prime }}_{l} & -{v^{\prime }}_{l}\times v_{s} & v_{s} \end{array}\right].$$

After calculating *R*, all pairs of two lines composing stop lines are transformed by homography *H* in Equation (3). Since the two lines of the correct pair should be parallel to each other after the transformation, the number of line pairs parallel to each other is counted as a consensus set. This procedure is iterated and *R_max_* that maximizes the number of consensus set is selected. Finally, the line pairs excluded from the consensus set of *R_max_* are identified as outliers and rejected. The roll angle is obtained by decomposing *R_max_*. After removing the outliers, the roll angle is refined using LM algorithm by minimizing the cost, *c_rs_* as

(7) $$c_{rs}=\sum_{i=1}^{N_{S}}\left|\left|tl_{i}-bl_{i}\right|-\left|tr_{i}-br_{i}\right|\right|,$$

where *N_S_* is the number of stop lines classified as inliers. *tl_i_*, *bl_i_*, *tr_i_*, and *br_i_* are shown in Figure 9b. *tl_i_* and *tr_i_* are the vertical locations of the intersection points between the top line of the *i*-th stop line and *x* = 0 and *x* = *w_max_*, respectively, and *bl_i_* and *br_i_* are the vertical locations of the intersection points between the bottom line of the *i*-th stop line and *x* = 0 and *x* = *w_max_*, respectively. *c_rs_* in Equation (7) is minimized when the top and bottom lines of the stop lines are parallel to each other.

**Figure 10:**
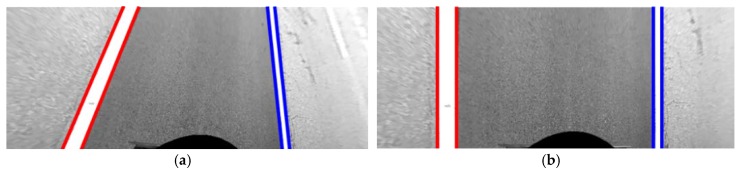
Images before and after the homography-based transformation. (**a**) Before the transformation; (**b**) After the transformation.

**Figure 11:**
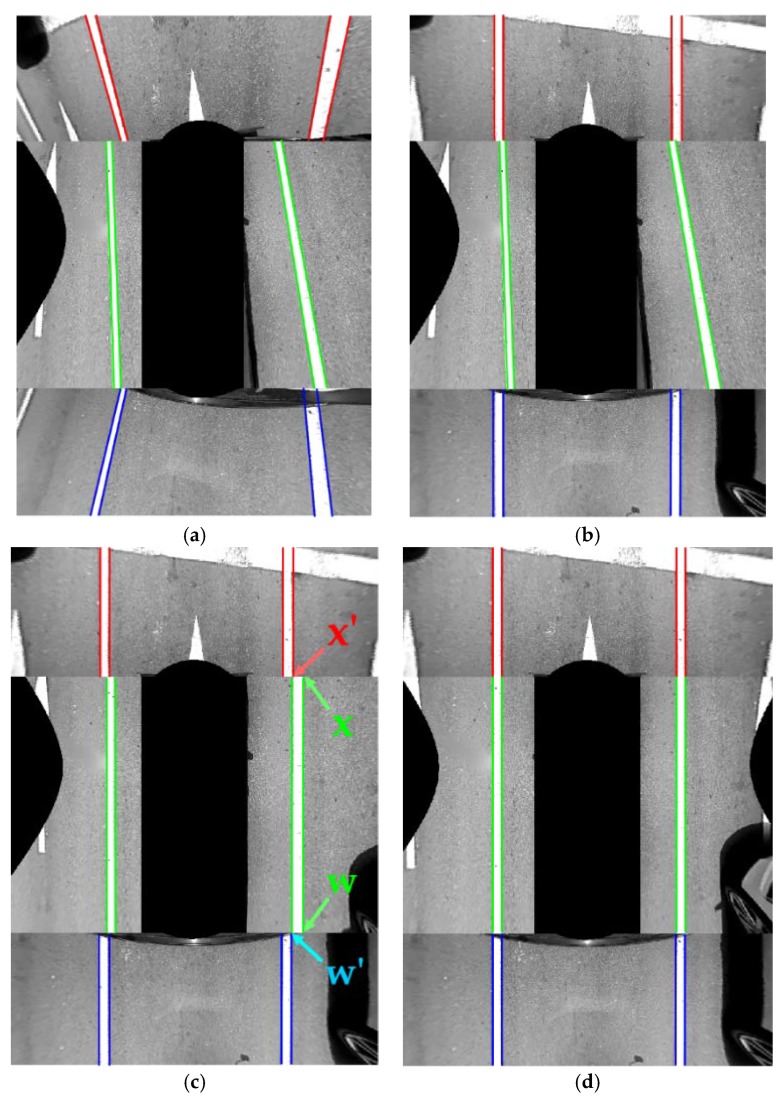
Resulting AVM images at consecutive calibration stages. (**a**) Before the calibration; (**b**) After calibrating the front and rear cameras; (**c**) After estimating the yaw and roll angles of two side cameras; (**d**) After calibrating all pitch, yaw, roll angles of four cameras. Red, blue, and green lines indicate the lane markings in the front, rear, and side cameras, respectively.

If the number of detected stop lines are insufficient, the roll angle is estimated by lane markings by assuming that the left and right lane markings detected in the same image have the same width. Since there are some cases where this assumption is not valid, this method first rejects the lane marking pairs whose widths are expected to be different to each other. To this end, the lane markings are first transformed by the homography, which is calculated by the vanishing point of the left and right lane markings via Equations (1)–(3). Figure 10a,b show the lane markings before and after the homography-based transformation, respectively. Since this homography transforms the vanishing point to the point at infinity, **p***_∞_* = [0 1 0]^T^, all lines composing lane markings become parallel to the vertical axis of the image. After the transformation, this method rejects pairs of left and right lane markings based on their width ratio using RANSAC. A pair of left and right lane markings detected in the same image is randomly selected, and the ratio between their widths is calculated. After that, the number of lane marking pairs whose width ratio is similar to this ratio is counted as a consensus set. This procedure is iterated, and the width ratio that maximizes the number of consensus set is selected. The lane marking pairs whose width ratios are different from the selected width ratio are classified as outliers and rejected. After the outlier removal, the roll angle is estimated using LM algorithm by minimizing the cost, *c_rl_* as

(8) $$c_{rl}=\sum_{i=1}^{N_{P}}\left|wl_{i}-wr_{i}\right|,$$

where *N_P_* is the number of pairs of left and right lane markings detected in the same images. *wl_i_* and *wr_i_* are the widths of the *i*-th lane marking pair. *wl_i_* is for the left lane marking and *wr_i_* for the right lane marking. Based on this approach, all pitch, yaw, and roll angles of both the front and rear cameras are estimated. Figure 11a shows the AVM image before the camera calibration, and Figure 11b shows the AVM image after calibrating the front and rear cameras. Red, blue, and green lines indicate the lane markings in the front, rear, and side cameras, respectively. It can be noticed that two lane markings in images of the front and rear cameras are upright, and have the same widths as in Figure 11b. This implicitly reveals that the angles of the front and rear cameras are correctly estimated. But, in Figure 11b, the lane markings in the images of the two side cameras are not correctly located because the two side cameras have not been yet calibrated.

### 5.2. Calibration of Left and Right Cameras

Once the front and rear cameras are calibrated, the two side cameras are then calibrated. Figure 8c,d show the pitch, yaw, and roll of the left and right cameras, respectively. The left and right cameras are separately calibrated using the same method. This method first estimates the yaw and roll angles of the side camera, and then estimates its pitch angle with the help of the calibration results of the front and rear cameras. The yaw and roll angles of the side camera are estimated by the same method used for estimating the pitch and yaw angles of the front and rear cameras in Section 5.1. That is, the yaw and roll angles of the side camera are obtained by finding the vanishing point via Equation (4) and minimizing the cost in Equation (5). However, in the case of the side camera, the pitch angle cannot be obtained by itself. This is because, unlike the front and rear cameras, only a single lane is observable in an image of the side camera. Figure 11c shows the AVM image after calibrating the yaw and roll angles of the two side cameras. The lane markings in the images of the two side cameras are upright because the yaw and roll angles have been calibrated. However, their locations and widths are not consistent with the lane markings in the images of the front and rear cameras. This is because the pitch angles of two side cameras cannot be calibrated by themselves. To overcome this limitation, this paper proposes an approach that estimates the pitch angles of the two side cameras with the help of the calibration results of the front and rear cameras.

The proposed method finds the pitch angle that makes the lane markings appearing across images of adjacent cameras properly connect to each other. This method has two assumptions: one is that some of the lane markings are simultaneously captured by adjacent cameras, and the other is that the detected lane markings are locally straight. Since it cannot be assumed that all lane markings detected in images of adjacent cameras at the same time correspond to each other, this method uses RANSAC to simultaneously find the corresponding lane markings and the pitch angle of the side camera. This method first randomly selects one line detected in an image of the side camera, and finds the lines detected in images of the front or rear camera at the same time. If there are lines detected in images of the side and front cameras at the same time, it is assumed that two lines correspond each other. The upper end point of the line detected in the side camera image is denoted as **x** = [*u* *v* 1]^T^, and the lower end point of the line detected in the front camera image is denoted as **x′** = [*u*’ *v*’ 1]^T^. The locations of **x** and **x′** are shown in Figure 11c. Those two points are related with the pitch angle, *ϕ*, of the side camera as

(9) $$x^{\prime }=KR_{\phi }K^{-1}x,$$

where *K* is the intrinsic parameters matrix of the virtual camera of the AVM system, which is predetermined when the AVM system is manufactured. *R_ϕ_* is a 3 × 3 rotation matrix induced by *ϕ*. If *K* moves from the right side to the left side in Equation (9), it is converted as

(10) $$\begin{array}{c} \underset{y^{\prime }}{\underset{︸}{K^{-1}x^{\prime }}}=R_{\phi }\underset{y}{\underset{︸}{K^{-1}x}}\\ y^{\prime }=R_{\phi }y \end{array}.$$

Since the vertical locations of **x** and **x**’ are the same as shown in Figure 11c, ***y*** and ***y*′** can be denoted as [*x* *y* 1]^T^ and [*x*′ *y* 1]^T^, respectively. Based on these notations, Equation (10) can be rewritten as

(11) $$\left[\begin{array}{c} x^{\prime }\\ y\\ 1 \end{array}\right]=\left[\begin{array}{ccc} \cos\phi & 0 & \sin\phi \\ 0 & 1 & 0\\ -\sin\phi & 0 & \cos\phi \end{array}\right]\left[\begin{array}{c} x\\ y\\ 1 \end{array}\right].$$

Since the second row of Equation (11) is meaningless, it can be simplified as

(12) $$\left[\begin{array}{c} x^{\prime }\\ 1 \end{array}\right]=\left[\begin{array}{cc} \cos\phi & \sin\phi \\ -\sin\phi & \cos\phi \end{array}\right]\left[\begin{array}{c} x\\ 1 \end{array}\right].$$

In Equation (12), [*x* 1]^T^ and [*x*′ 1]^T^ are related with a 2 × 2 rotation matrix. Thus, the pitch angle, *ϕ* of the side camera can be calculated by using Procrustes analysis [31] as

(13) $$\phi =-\tan^{-1}\left(\frac{x-x^{\prime }}{xx^{\prime }+1}\right).$$

The same procedure can be used for estimating the pitch angle, *ϕ* using the lower end point of the line detected in the side camera image, **w**, and the upper end point of the line detected in the rear camera image, **w′**. The locations of **w** and **w′** are shown in Figure 11c. Once *ϕ* is estimated, all the lines detected in images of the side camera, **l***_s_* are transformed by homography, *H_ϕ_* as

(14) $${l^{\prime }}_{s}=H_{\phi }^{-T}l_{s}={\left(KR_{\phi }K^{-1}\right)}^{-T}l_{s}.$$

After the transformation, the number of line correspondences between the images of adjacent cameras is counted as a consensus set. This procedure is iterated, and the pitch angle that produces the largest number of consensus set is selected as the pitch angle of the side camera. In addition, the line correspondences included in the largest consensus set are classified as correct corresponding lines between adjacent cameras. The pitch angle estimated by RANSAC, is refined using LM algorithm by minimizing the cost, *c_sp_*, as

(15) $$c_{sp}=\sum_{i=1}^{N_{C}}\left|u_{i}-{u^{\prime }}_{i}\right|,$$

where *u_i_* and *u_i_*’ are the horizontal locations of the *i*-th line correspondence after the transformation using Equation (14). *N_C_* is the number of line correspondences. *c_sp_* is minimized when line correspondences detected in images of adjacent cameras are exactly connected to each other. Figure 11d shows the AVM image produced by the calibration results of all four cameras. It can be seen that the lane markings in images of adjacent cameras are properly connected to each other.

### 5.3. Parameter Co-Refinement of Four Cameras

Once all four cameras of the AVM system are initially calibrated using the two-step approach explained in Section 5.1 and Section 5.2, the proposed method simultaneously refines all angles of four cameras using LM algorithm by minimizing the cost, *c_total_*, that consists of *c_py_* in Equation (5), *c_rs_* in Equation (7), *c_rl_* in Equation (8), and *c_sp_* in Equation (15) as

(16) $$c_{total}=\{\begin{array}{cc} c_{py,front}+c_{py,rear}+c_{py,left}+c_{py,right}+c_{rs,front}+c_{rs,rear}+c_{sp,left}+c_{sp,right}, & N_{S}>T\\ c_{py,front}+c_{py,rear}+c_{py,left}+c_{py,right}+c_{rl,front}+c_{rl,rear}+c_{sp,left}+c_{sp,right}, & otherwise \end{array},$$

where the second subscription of *c* indicates the camera used for calculating the cost. *N_S_* and *T* are the number of stop lines and the predetermined minimum number of stop lines, respectively. The proposed method simultaneously refines the initial calibration results of four cameras, not only using the lane markings detected in images taken from individual cameras, but also using the corresponding lane markings matched between adjacent cameras. This approach can make the produced AVM images more seamless. The corresponding lane markings have been obtained during the RANSAC-based pitch angle estimation of the side cameras in Section 5.2.

## 6. Parameter Selection

If the proposed calibration procedure is applied to a long image sequence, it requires a huge amount of computational resources (memory and time) because a huge number of lane markings should be stored and processed. To alleviate this limitation, this procedure is conducted multiple times in the case of a long image sequence. That is, the camera parameters are repeatedly estimated whenever a sufficient number of lane markings are gathered. When using this approach, multiple parameter sets are obtained. A parameter set includes 12 camera angles (three angles for each camera), so that it can be considered as a 12-dimensional vector. To find the most appropriate parameter set and prevent it from being overfitted to a specific place, this paper selects the medoid (**a′**) of multiple parameter sets (**a**_1_, **a**_2_, …, **a***_NA_*) as

(17) $$a^{\prime }=\underset{b\in \left\{a_{1},a_{2},\cdots ,a_{N_{A}}\right\}}{\arg\min}\sum_{i=1}^{N_{A}}d\left(a_{i},\;b\right),$$

where *N_A_* is the number of parameter sets, and *d* (**a***_i_*, **b**) indicates the Euclidean distance between two parameter sets (**a***_i_* and **b**).

## 7. Experiments

### 7.1. Experimental Setup

**Figure 12:**
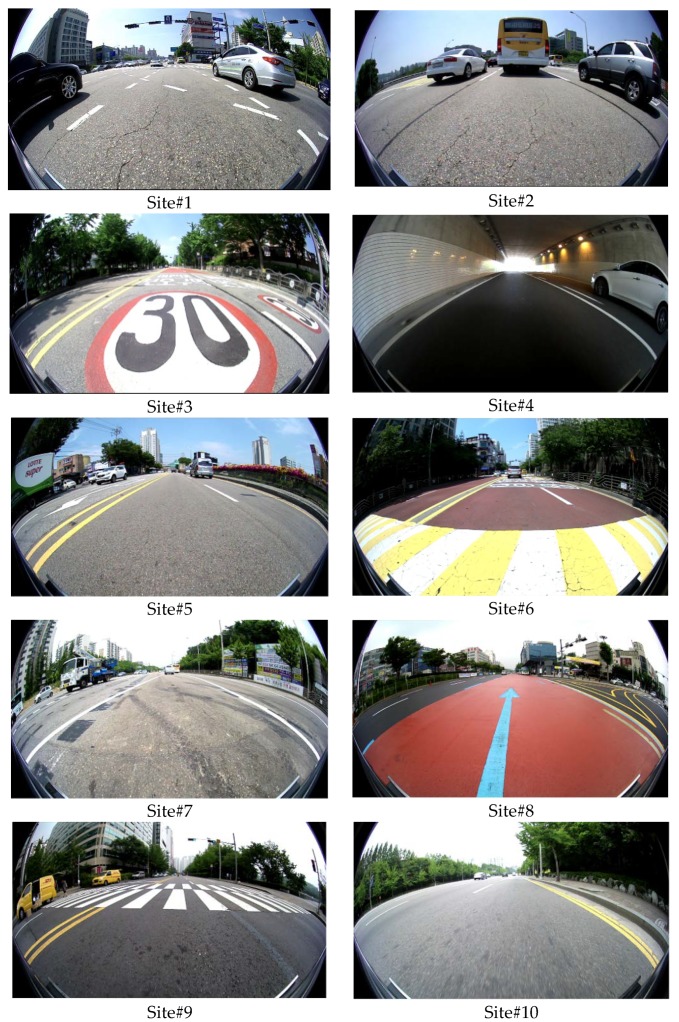
Example images of the test dataset taken at 10 different sites.

The test dataset was acquired by the AVM system mounted on an off-the-shelf vehicle, a Hyundai Genesis G80 [32]. The AVM system consists of four fisheye cameras located at the centers of the front and rear bumpers, and under two side view mirrors, as shown in Figure 1. The resolution, field-of-view, and acquisition frequency of each fisheye camera are 1280 × 720 pixels, 190 degrees, and 30 frames per second, respectively. The test dataset was captured at 10 different sites for 116 min. Figure 12 shows example images included in the test dataset. Only the images taken by the front camera of the AVM system are presented. As shown in this figure, the test dataset was acquired in various real driving situations including congested, uncongested, wide, and narrow roads.

In order to quantitatively evaluate the performance of the proposed method, the ground truth angles of four cameras are necessary. To obtain them, the intrinsic parameters of four cameras are first calibrated using the method suggested in [33], and then the extrinsic parameters are estimated using a sophisticatedly designed calibration pattern shown in Figure 2. The camera angles obtained by this procedure are considered as the ground truth camera angles. When applying the proposed method to the test dataset, five degrees of noise with random signs are added to 12 ground truth camera angles (three angles for each camera). Those noisy camera angles are called the initial camera angles, and the proposed method conducts the camera calibration starting from the initial camera angles. Once the proposed method estimates three angles per each camera, those angles are compared with the corresponding ground truth camera angles. Since there are too many camera angles (12 angles), it is difficult to understand the experimental results at a glance if all of their errors are presented. Thus, this paper suggests a single measure called an average camera angle error, which is obtained by taking the average of differences between the estimated camera angles and corresponding ground truth camera angles. Detailed errors on pitch, roll, and yaw will be presented later when summarizing the performance evaluation.

### 7.2. Performance Evaluation

Table 1 shows the average camera angle error of the proposed two-step approach explained in Section 5.1 and Section 5.2 at 10 different sites. The two-step approach consists of the front and rear camera calibration followed by the left and right camera calibration. The results shown in Table 1 are the errors before applying the parameter co-refinement of four cameras explained in Section 5.3. This table reveals that the proposed two-step approach can estimate the angles of the front and rear cameras where both left and right lanes are observable as well as the angles of the left and right cameras where only one of two lanes is observable. The two-step approach without the parameter co-refinement gives 0.48 degrees of the average camera angle error at 10 different sites. It can be seen that the errors of the left and right cameras are relatively larger than those of the front and rear cameras. This is because the front and rear cameras are calibrated using both left and right lanes, but the left and right cameras are calibrated using only one of two lanes. This means that the amount of information used for calibrating the left and right cameras is less than that of the front and rear cameras. It is considered that the difference of lane marking information causes the difference of the average camera angle errors.

**Figure 13:**
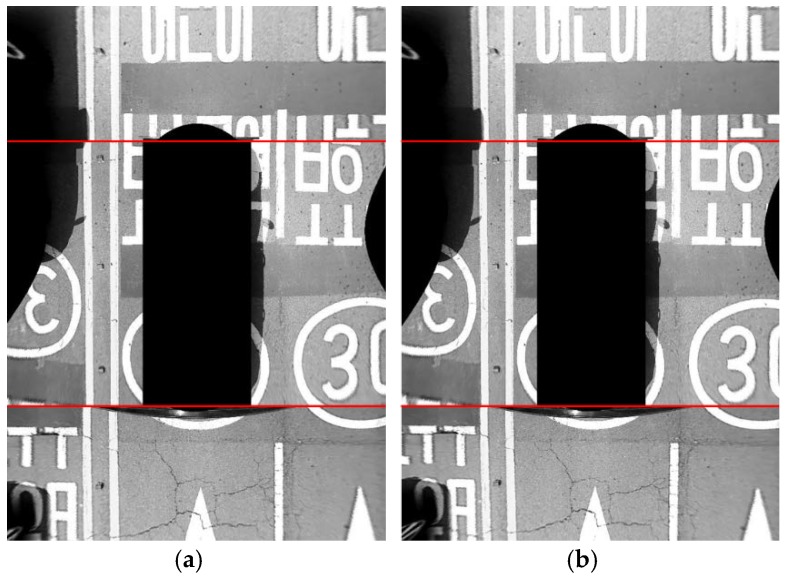
Resulting AVM images (**a**) without the parameter co-refinement, and (**b**) with the parameter co-refinement. Red lines indicate image boundaries.

Table 2 shows the average camera angle error at 10 different sites after applying the parameter co-refinement of four cameras, which is explained in Section 5.3. The parameter co-refinement procedure simultaneously refines the angles of four cameras estimated by the two-step approach based on the corresponding lane markings appearing across images of adjacent cameras. The proposed method with the parameter co-refinement gives 0.41 degrees of the average camera angle error at 10 different sites. It can be seen that the parameter co-refinement procedure reduces the average camera angle error by 15% (0.08 degrees) compared to the result without this procedure. The co-refinement procedure not only quantitatively reduces the camera angle error, but also qualitatively increases the quality of the AVM image. This is because the co-refinement procedure simultaneously refines the angles of four cameras in the direction that the corresponding lane markings appearing across images of adjacent cameras are smoothly connected. Since drivers cannot directly measure the camera angle error, they are likely to judge the quality of the AVM image by checking if images taken from adjacent cameras are smoothly connected at their boundaries. Figure 13a,b show the resulting AVM images without and with the parameter co-refinement procedure, respectively. It can be seen that the quality of the AVM image with the co-refinement (Figure 13b) is superior to that without it (Figure 13a) based on the fact that the road markings captured from adjacent cameras are smoothly connected to each other at the image boundaries (red lines). Since the AVM image is created for the purpose of showing it to the driver, it is important to find not only the parameters with small average camera angle error, but also the parameters that smoothly stitch the images taken by adjacent cameras.

Table 3 shows the average camera angle error at 10 different sites after applying both the parameter co-refinement and parameter selection explained in Section 6. The parameter selection procedure selects a set of camera angles by finding the medoid of multiple camera angle sets obtained from a long image sequence. The proposed method with both the parameter co-refinement and parameter selection gives 0.31 degrees of the average camera angle error at 10 different sites. It can be seen that the parameter selection procedure reduces the average camera angle error by 24% (0.10 degrees) compared to the result without this procedure. It reveals that the parameter selection procedure not only reduces computational resources by separating a long image sequence and processing them individually, but also improves the camera angle estimation performance by decreasing the dependency on a specific place. In Table 3, the errors of the left and right cameras are still shown to be larger than those of the front and rear cameras. However, it can be seen that the difference between the errors of the front and rear cameras and the errors of the left and right cameras are remarkably reduced compared to the results in Table 1 and Table 2.

Table 4 summarizes the average camera angle errors of three different experimental settings shown in Table 1, Table 2 and Table 3 with detailed pitch, roll, and yaw angle errors. Among three settings, the one with both the co-refinement and parameter selection gives the best performance. In this setting, the left and right cameras give higher errors than the front and rear cameras. In more detail, in terms of the roll and yaw angles, the left and right cameras have a similar amount of errors to the front and rear cameras. This means the error difference mostly comes from the pitch angles. In the last row of Table 4, it can be found that the pitch angle errors of the left and right cameras are higher than those of the front and rear cameras. In case of the front and rear cameras, both left and right lane markings are captured, so that their pitch angles can be estimated by their own lane information as described in Section 5.1. However, in the case of the left and right cameras, only one of two lane markings is captured, so that their pitch angles cannot be estimated by their own lane information, as explained in Section 5.2. Due to this limitation, this paper utilizes the approach that calibrates the pitch angles of the left and right cameras with the help of the calibration results of the front and rear cameras. This means that the pitch angles of the left and right cameras are more severely affected by the errors of the front and rear cameras, compared to the roll and yaw angles that can be estimated without the help of the calibration results of the front and rear cameras. This is considered to be the reason that makes the pitch angle have a higher error than the roll and yaw angles in the case of the left and right cameras.

**Figure 14:**
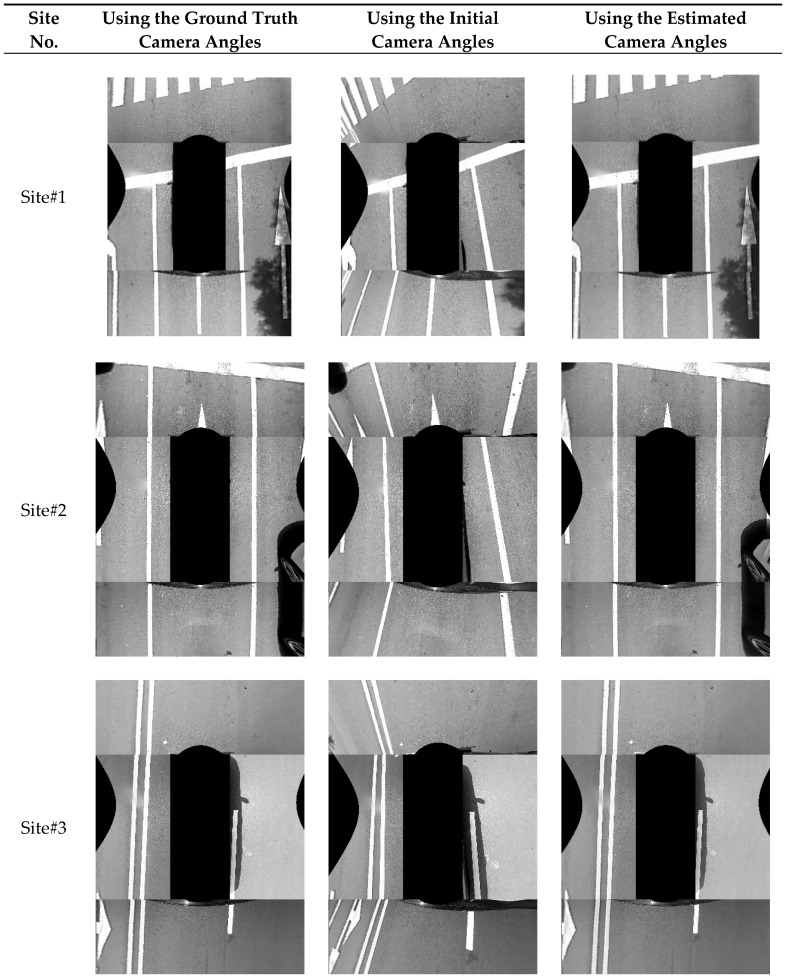

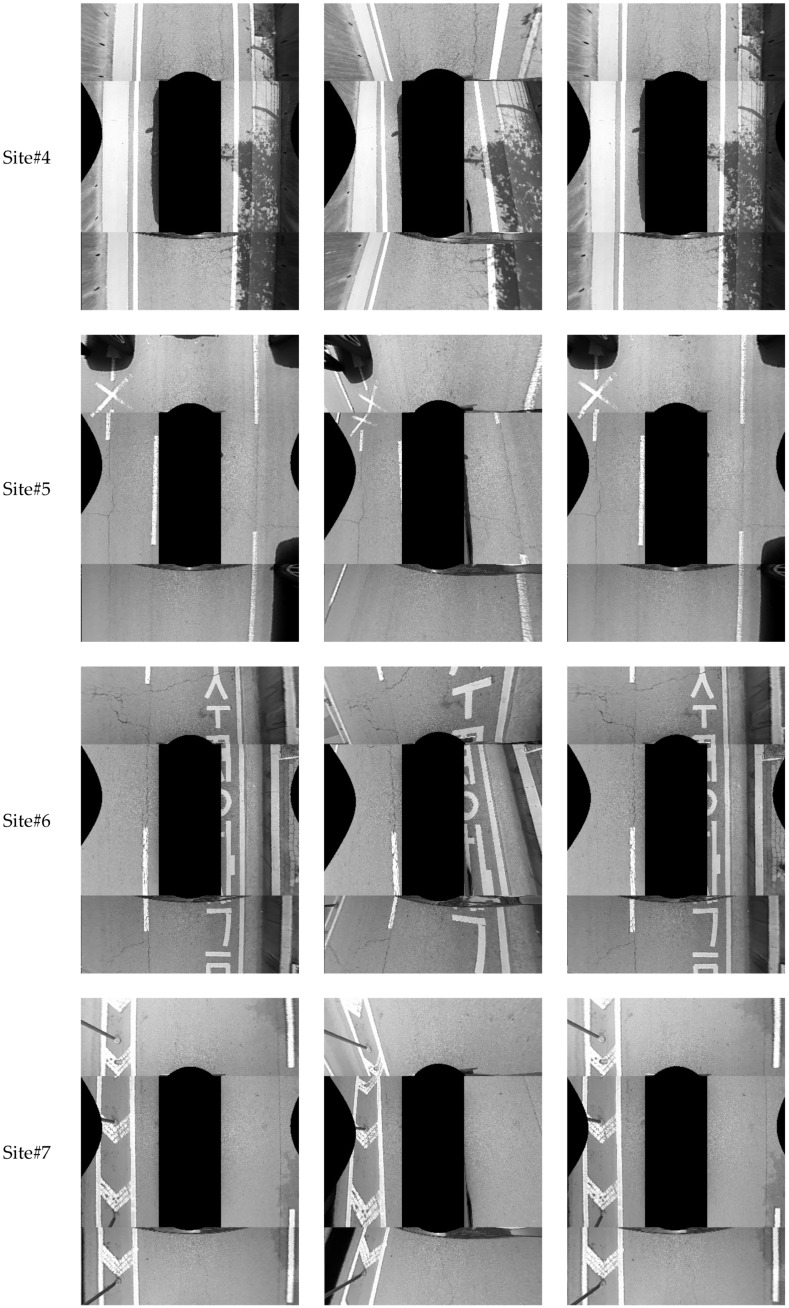

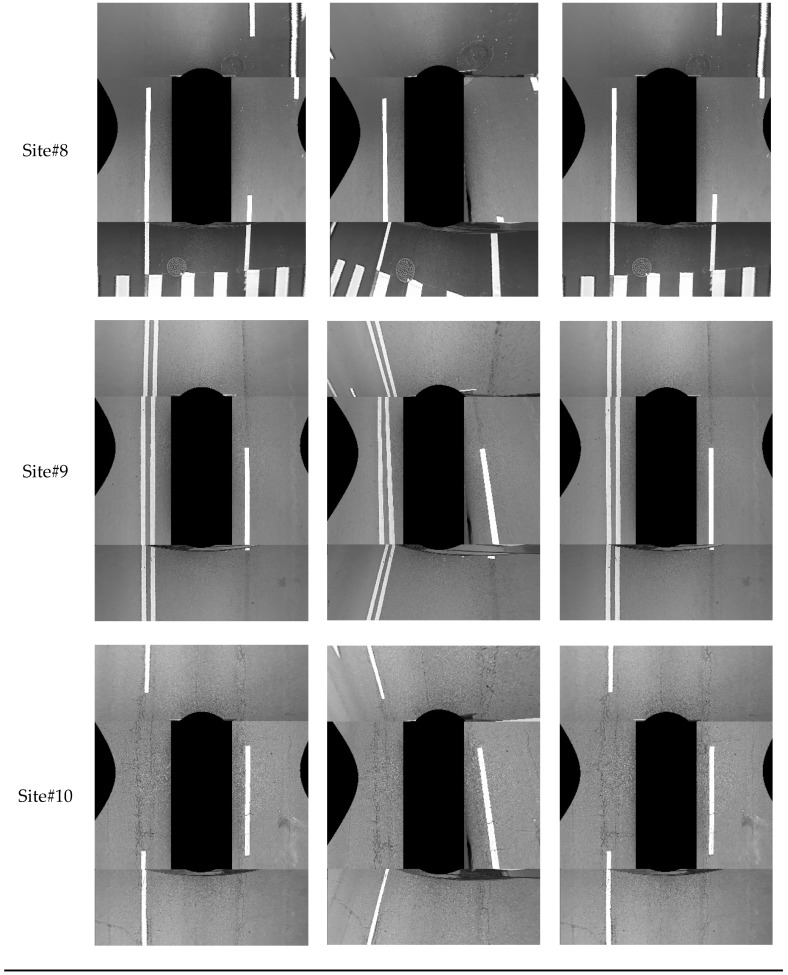
Resulting AVM images at 10 different sites. (**left**) AVM images generated by the ground truth camera angles; (**middle**) AVM images generated by the initial camera angles; (**right**) AVM images generated by the camera angles resulting from the proposed method.

Figure 14 shows the resulting AVM images at 10 different sites. In this figure, the left, middle, and right columns show the AVM images generated by the ground truth camera angles, initial camera angles with five degrees of noise with random signs, and the camera angles estimated by the proposed method, respectively. At this point, the proposed method indicates the two-step approach with both the co-refinement and parameter selection. In the AVM images produced by the proposed method, it can be seen that the road markings including lanes, stop lines, arrows, letters, etc., are well connected across images of adjacent cameras. In addition, it can be noted that the AVM images produced by the proposed method are quite similar to those generated by the ground truth camera angles. Based on these results, it can be said that the proposed method shows a promising performance for estimating the angles of four cameras composing the AVM system.

Note that there are mainly two cases where even the AVM images generated by the ground truth camera angles include some discontinuity at image boundaries. The first case is shown in the AVM images at Site#4 and Site#6 of Figure 14. In the left half region of the AVM image at Site#4, and the right half region of the AVM image at Site#6, there are some object regions that include large discontinuities. These discontinuities are not because the camera angles are incorrectly estimated, but because those objects (curbs) have different heights from the ground. In AVM images, only the objects whose heights are the same as the ground are connected across the images of adjacent cameras. The second case is shown in the AVM images at Site#7 and Site#8 of Figure 14. In the left-most lane, marking at Site#7 and the right-most lane marking at Site#8, there are some discontinuities, even though these markings are drawn on the ground. These discontinuities are not because of the incorrect camera calibration, but because of the non-flat ground. One of the most fundamental assumptions of the AVM system is that the ground is flat. This assumption is sometimes invalid in the area distant from the ego-vehicle, and this makes some discontinuities in the AVM image. Note that these two cases of discontinuities are not due to the camera angle estimation error but a fundamental characteristic of the AVM system.

### 7.3. Execution Time

Table 5 shows the execution time for four main modules of the proposed method. These times were measured on an Intel Core i7-2600 CPU using only a single core. Note that among four modules, only the lane marking detection module is required to be processed in real time. The other three modules need to be processed only once after a sufficient number of lane markings are gathered. In Table 5, the lane marking detection module requires 26.12 ms to process four images acquired from four cameras of the AVM system, which means that this module can process more than 30 frames per second in real time. The other three modules require a total execution time of 101.76 ms. Since those modules need to be processed only once after gathering lane markings, their execution times do not hinder the proposed method from operating in real time.

### 7.4. Comparison with Previous Methods

As aforementioned in Section 2, the previous methods suggested for calibrating vehicle-mounted cameras can be categorized into three approaches: calibration pattern-based, interest point-based, and lane marking-based. Table 6 shows the comparison of the previous and proposed methods from the viewpoint of the AVM system calibration. The calibration pattern-based methods can consider the inter-camera relationship and handle side cameras. However, they are inconvenient because the driver must visit a specific place where the pattern is installed, and it cannot be conducted in a natural driving situation. The interest point-based methods do not require the driver to visit a specific place and are applicable to side cameras. However, the vehicle must travel at a very low speed to stably track interest points. In addition, it is hard to obtain point correspondences between images of adjacent cameras because overlapping areas are severely distorted in the case of the AVM system. This makes it difficult to consider the inter-camera relationship. The lane marking-based methods do not require the driver to visit a specific place and can be applied at both low and high vehicle speeds. However, the previous lane marking-based methods cannot consider the inter-camera relationship because they do not utilize the corresponding lane markings that appear across images of adjacent cameras. Furthermore, those methods cannot handle side cameras because they assume that both left and right lane markings are observable in a single camera. In contrast to those methods, the proposed method can consider the inter-camera relationship by using corresponding lane markings across images of multiple cameras, and calibrate all four cameras including the side ones by using the two-step approach.

Unfortunately, since there is no previous method that can calibrate all four cameras of the AVM system in a natural driving situation, including low and high speeds, it is impossible to conduct quantitative performance comparison of the previous and proposed methods with the same dataset. However, the comparison summarized in Table 6 clearly explains that the proposed method is superior to the other previous methods in terms of automatic AVM system calibration.

## 8. Conclusions

This paper proposes a novel and practical method that calibrates the AVM system in a fully automatic manner using lane markings. The proposed method has the following advantages: First, it is applicable to a natural driving situation where the vehicle travels at both low and high speeds. Second, it calibrates not only the front and rear cameras but also the left and right cameras where only one of two lane markings is captured. Last, it considers the inter-camera relationship using the corresponding lane markings across images of adjacent cameras to produce seamless AVM images. The proposed method was evaluated by the image sequences taken in various real driving conditions and showed a promising performance along with a real-time processing capability. This method is expected to improve the driver’s convenience by automatically adjust the AVM system in the case of the posture changes of the cameras.

## Figures and Tables

**Table 1:** Average Camera Angle Error without Parameter Co-Refinement (Degree).

Site No.	Front Camera	Rear Camera	Left Camera	Right Camera	Overall
1	0.42	0.40	0.70	0.54	0.51
2	0.47	0.63	0.73	1.16	0.75
3	0.20	0.46	0.22	0.66	0.39
4	0.26	0.39	0.53	0.47	0.41
5	0.38	0.38	0.65	0.52	0.48
6	0.33	0.33	0.51	0.37	0.38
7	0.39	0.43	0.69	0.71	0.56
8	0.36	0.34	0.69	0.46	0.46
9	0.33	0.37	0.52	0.45	0.42
10	0.28	0.35	0.52	0.46	0.40
**Overall**	**0.34**	**0.41**	**0.58**	**0.58**	**0.48**

**Table 2:** Average Camera Angle Error with Parameter Co-Refinement (Degree).

Site No.	Front Camera	Rear Camera	Left Camera	Right Camera	Overall
1	0.37	0.25	0.53	0.48	0.41
2	0.44	0.38	0.53	0.92	0.57
3	0.20	0.24	0.34	0.31	0.27
4	0.26	0.30	0.73	0.41	0.42
5	0.34	0.27	0.68	0.39	0.42
6	0.35	0.24	0.65	0.43	0.42
7	0.33	0.24	0.65	0.53	0.44
8	0.34	0.24	0.71	0.36	0.41
9	0.34	0.18	0.60	0.37	0.37
10	0.34	0.19	0.55	0.37	0.36
**Overall**	**0.33**	**0.25**	**0.60**	**0.46**	**0.41**

**Table 3:** Average Camera Angle Error with Parameter Co-Refinement and Parameter Selection (Degree).

Site No.	Front Camera	Rear Camera	Left Camera	Right Camera	Overall
1	0.25	0.33	0.25	0.43	0.31
2	0.31	0.34	0.14	0.68	0.37
3	0.20	0.21	0.22	0.27	0.23
4	0.21	0.34	0.57	0.12	0.31
5	0.33	0.28	0.50	0.37	0.37
6	0.39	0.22	0.50	0.24	0.34
7	0.19	0.18	0.50	0.06	0.23
8	0.26	0.25	0.51	0.22	0.31
9	0.34	0.17	0.37	0.43	0.33
10	0.33	0.15	0.37	0.35	0.30
**Overall**	**0.28**	**0.25**	**0.39**	**0.32**	**0.31**

**Table 4:** Quantitative Performance Comparison with Different Experimental Settings (Degree).

Experimental Setting	Front Camera	Rear Camera	Left Camera	Right Camera	Overall
	Pitch/Roll/Yaw	Pitch/Roll/Yaw	Pitch/Roll/Yaw	Pitch/Roll/Yaw	Pitch/Roll/Yaw
**W/O** Co-Refinement	0.34	0.41	0.58	0.58	0.48
**W/O** Parameter Selection (Table 1)	0.42/0.31/0.30	0.50/0.40/0.32	0.88/0.45/0.40	1.18/0.28/0.28	0.74/0.36/0.33
**W**/Co-refinement	0.33	0.25	0.60	0.46	0.41
**W/O** Parameter Selection (Table 2)	0.46/0.22/0.31	0.24/0.24/0.28	0.93/0.42/0.45	0.80/0.31/0.26	0.61/0.30/0.33
**W**/Co-Refinement	0.28	0.25	0.39	0.32	0.31
**W**/Parameter Selection (Table 3)	0.42/0.18/0.24	0.28/0.19/0.27	0.68/0.21/0.28	0.51/0.24/0.20	0.47/0.20/0.25

**Table 5:** Execution time (ms).

Module	Time
Lane marking detection	26.12
False lane marking removal	22.02
Parameter estimation	79.69
Parameter selection	0.05

**Table 6:** Comparison of the previous and proposed methods.

Method		Driver’s Convenience	High Speed Condition	Inter-Camera Relationship	Side Camera Handling
Calibration pattern-based methods		Poor	Poor	Good	Good
Interest point-based methods		Good	Poor	Fair	Good
Lane marking-based methods	Previous methods	Good	Good	Poor	Poor
	**Proposed method**	**Good**	**Good**	**Good**	**Good**
